# Epitope-tagged yeast strains reveal promoter driven changes to 3′-end formation and convergent antisense-transcription from common 3′ UTRs

**DOI:** 10.1093/nar/gkv1022

**Published:** 2015-10-19

**Authors:** Angavai Swaminathan, Traude H. Beilharz

**Affiliations:** Development and stem cells Program, Monash Biomedicine Discovery Institute and Department of Biochemistry and Molecular Biology, Monash University, Melbourne, Victoria 3800, Australia

## Abstract

Epitope-tagging by homologous recombination is ubiquitously used to study gene expression, protein localization and function in yeast. This is generally thought to insulate the regulation of gene expression to that mediated by the promoter and coding regions because native 3′ UTR are replaced. Here we show that the 3′ UTRs, *CYC1* and *ADH1*, contain cryptic promoters that generate abundant convergent antisense-transcription in *Saccharomyces cerevisiae*. Moreover we show that aberrant, truncating 3′ –end formation is often associated with regulated transcription in TAP-tagged strains. Importantly, the steady-state level of both 3′ –truncated and antisense transcription products is locus dependent. Using TAP and GFP-tagged strains we show that the transcriptional state of the gene-of-interest induces changes to 3′ –end formation by alternative polyadenylation and antisense transcription from a universal 3′ UTR. This means that these 3′ UTRs contains plastic features that can be molded to reflect the regulatory architecture of the locus rather than bringing their own regulatory paradigm to the gene-fusions as would be expected. Our work holds a cautionary note for studies utilizing tagged strains for quantitative biology, but also provides a new model for the study of promoter driven rewiring of 3′ –end formation and regulatory non-coding transcription.

## INTRODUCTION

The plasticity of the *Saccharomyces cerevisiae* genetic model is beloved by all who have worked with it. Easy transformation and efficient homologous recombination has meant that researchers working with baker's yeast were among the first to embrace designer genomic knock-out (1) and knock-in (2,3) collections. These features of easy genetic manipulation have been exceptionally powerful tools in the discovery of global –network based trends and specific gene-by-gene dissection of eukaryotic cell biology. However, with recent technological advances we have also learned that higher-order organisation of gene expression is controlled by complex three-dimensional chromatin structures that regulate sense and antisense transcription, regulatory transcription and 3′ -end formation (4). For example, the looping interactions between the transcription initiation and 3′ -end formation machineries are responsible for the choice of promoter directionality (5). High density tiling microarrays (6–8) and RNA-seq experiments (9,10) now all show the ubiquity of both stable and transient non-coding RNA in eukaryotes. Non-coding transcripts fall into a number of categories (CUTs, SUTs, XUTs, etc.) depending on their metabolism. It is important to note that these designations are likely context dependent, with most XUTs (Xrn1-sensitive unstable transcripts), for example becoming SUTs (Stable Unannotated Transcripts) in the presence of lithium (11). Annotated SUTs and CUTs become cyclically expressed with inverse phasing to their overlapping coding transcripts within the cell-cycle (6). Together such data highlight how much we still need to learn about the functional metabolism of non-coding RNA.

The literature contains early examples of regulatory control on forward transcription imposed by convergent antisense transcription. For example, the prolonged storage of yeast cells at 4°C induces heritable expression of an antisense RNA that suppresses expression of the sense mRNA *PHO84* (12). More recently, the physical and mechanistic link between the initiation of transcription, 3′ -end formation, antisense transcription and chromatin architecture is clearly emerging as a widespread mechanism of gene silencing (13,14). At first, the mechanism of regulation by convergent transcription was interpreted as interference by transcriptional collision (15). This is clearly an important part to the story, but the fact that RNAi can be reconstituted in budding yeast (14,16) means that RNA duplexes of overlapping sense and antisense pairs must exist long enough to act as substrates in these systems, and therefore pose a further opportunity for regulation either in the nucleus or possibility also during cytoplasmic translation. Moreover, since antisense regulation by overlapping 3′ UTR can be enacted by ectopic expression of antisense RNA (*in trans*) it means that collision is not the only mechanism of regulatory control imposed by non-coding antisense RNA (17).

More than 30% of yeast transcripts show evidence for 3′ -end overlap (18–20). The absence of clear adenylation motifs beyond simple U or AU-rich sequences, and an abundance of micro-heterogeneity around the adenylation site in yeast ((19) and T. Beilharz unpublished observation), suggests that perhaps a minimal requirement for cleavage and adenylation is the presence of an accessible nucleosome free region, a feature also required for initiation of transcription (8). Here we document how standard genetic manipulation can result in unexpected antisense transcription and 3′ -end formation in *S. cerevisiae*, generated inadvertently by researchers through introduction of cryptic cleavage and adenylation signals and 3′ UTR located promoters. We show that the *CYC1* 3′ UTR, ubiquitously utilized in ectopic expression plasmids generates abundant antisense transcripts, and that epitope tagging with the localization tag GFP, at native genomic loci, can be associated with convergent antisense transcription from the *ADH1* 3′ UTR. Moreover, we show that the Tandem Affinity Purification (TAP) tag is prone to truncating cleavage and adenylation. In these genetically modified epitope-tagged strains, both aberrant 3′ -end formation and activation of 3′ UTR cryptic promoters is locus specific and dependent on the transcriptional state of the local genetic landscape. Together, our data provide a practical caution with regards to the use of expression plasmids and tagged strains for quantitative studies, but also conceptually promote the idea that 5′ driven regulation imposes reinforcing regulatory control over 3′ UTR dynamics.

## MATERIALS AND METHODS

### *Saccharomyces cerevisiae* culture

For plasmid expression, BY4741 (*MATa his3Δ0 leu2Δ0 met15Δ0 ura3Δ0*) cells were grown in rich selective media (2% raffinose, 0.67% yeast nitrogen base without amino acids and –ura drop-out mix) to exponential phase (OD_600_∼0.6). Transcription of Galactose (GAL) regulated genes was induced by addition of galactose (2%) and harvested after 1 h, snap frozen and stored at −80°C. For TAP-tagged strains, the cells were grown in YPEG (2% peptone; 1% yeast extract, 3% ethanol and 2% glycerol) to an OD_600_ of ∼0.6 and harvested for RNA analysis. For GFP-tagged strains the cells were grown either in YPD (2% peptone; 1% yeast extract, 2% glucose) or YPE (2% peptone; 1% yeast extract, 2% ethanol). Additional mutations were introduced into GFP and TAP tagged strains by homologous recombination of deletion cassettes re-amplified from confirmed mutant strains from the yeast deletion collection. The pGal plasmid was generated from a pRS423 backbone plasmid and 822 bases of the *GAL1* promoter, a was a gift from Dr G. Perrone (University of Western Sydney, Australia). The full-length open reading frame of *NGL3* was amplified from genomic DNA (see Supplemental Table S1 for primers) and ligated both in the forward and in the reverse orientation into the Bam HI site of this plasmid.

### RNA and protein analyses

Total RNA from yeast cells was prepared according to the hot phenol method. The ePAT and TVN-PAT assays were performed exactly as previously described (21). To capture longer 3′ RACE products (>500 bp) extension times were increased to 1 min, additional noise amplicons introduced by this are indicated (*) on the gel images. Note that because both ePAT and TVN-PAT assays depend on adenylation, non-coding RNA are sometimes variably detected between conditions. We sometimes detected antisense transcripts from the TAP cassette, but this was too variable to report with confidence for the three strains we tested. All data presented are representative of at least 3 independent experiments. To determine the exact adenylation sites, the PCR products from ePAT reactions were cloned using the Topo TA kit (Invitrogen) according to the manufacturer's procedures and Sanger sequenced using the Big-dye v3.1 (Applied Biosystems). The sequencing was performed at the Monash Micromon Facility. Reverse transcription reactions for qPCR was performed as previously described (22) using 500 ng of total RNA of interest and an additional 500 ng of HeLa (human cancer cell) RNA as spike-in normalizer. Detection of genes of interest was by Fast-Start universal SYBR Green Master (Roche) using LightCycler® 480 (Roche). Analysis was performed using linReg software (23). Data were normalized first to the level of human GAPDH to normalize for technical reproducibility of reverse transcription, followed by normalization to *S. cerevisiae ACT1* to normalize for RNA input. Primers are detailed in Supplemental Table S1 as are the results of sanger sequencing.

Flow cytometry was performed as previously described (24) with the strains grown to saturation in rich media, and analysed for GFP fluorescence using LSR IIa (BD Biosciences), and flowJo software. Total protein extracts were prepared as previously described (25), and immunoblots were probed with either a rabbit polyclonal antibody recognizing Hxk1/2 (gift from Trevor Lithgow) or a monoclonal anti-GFP antibody (Roche).

## RESULTS

### The *CYC1* terminator generates abundant antisense transcripts

**Figure 1.**
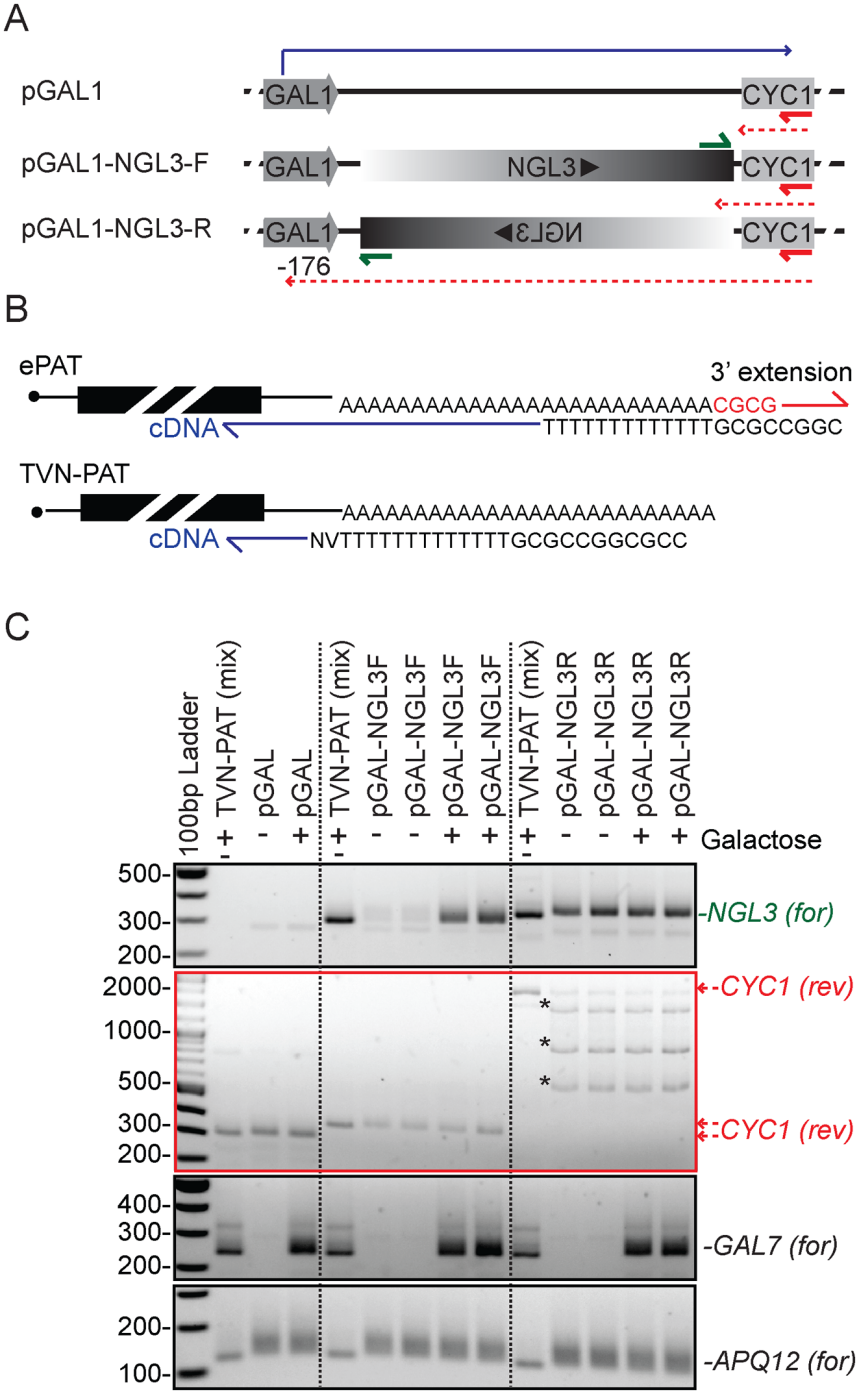
Abundant antisense transcription emanates from the CYC1 3′ UTR. (**A**) Schematic illustration the pGal expression plasmids. The *pGAL1-NGL3-F* plasmid contains the full open reading-frame of *NGL3*. *pGAL1-NGL3-R* is identical except that the *NGL3* gene is oriented in reverse complement. The solid blue line and arrowhead indicate the correct forward transcript initiation and 3′ -end processing. The dashed red line and arrows indicate the potential antisense transcripts emanating from the *CYC1* 3′ UTR. Green and red arrows indicate the primers annealing to cDNA generated from sense or antisense RNA, respectively. (**B**) The ePAT approach generates a DNA-tag at the 3′ end of adenylated RNA by Klenow mediated extension with dNTPs using a DNA oligo template. The tag is then used to prime reverse transcription. By this approach 3′ RACE products include the full poly(A)-tail. The TVN-PAT reaction utilizes 3′ variable bases in the RT-primer to lock reverse transcription to the junction between the 3′ UTR and the poly(A)-tail and thus indicates amplicon length with the shortest possible poly(A)-tail (21). (**C**) *NGL3* is expressed in both the forward and reverse orientation. Biological replicates of strains harbouring the empty plasmid, the *pGAL-NGL3-F* and *pGAL-NGL3-R* plasmids were grown ±1 h of galactose induction and subject to the ePAT assay. The TVN-PAT assay was performed on pooled samples (±Gal). Amplicons generated by the *NGL3* primer (top pane;l 25 cycles of PCR amplification) indicate that *NGL3* mRNA is abundantly expressed after galactose induction. However, this primer also generates similarly abundant amplicons when *NGL3* is in the reverse orientation, and this expression is independent of galactose driven transcriptional induction. Note the endogenous *NGL3* transcript is expressed at low levels in the culture conditions utilised, but is visible as a faint band below amplicons from the plasmid driven transcripts that utilise the *CYC1* 3′ UTR. Amplifications using the *CYC1* reverse primer show that each plasmid generates RNA in the antisense direction (red labelling indicating reverse orientation). The *pGAL1* empty and *pGAL1-NGL3-F* plasmid generate antisense transcripts (∼300 and ∼330 bp); the *NGL3-R* is transcribed in full and terminates with cleavage and adenylation within the GAL1 promoter region (24 cycles of amplification). * indicate non-specific amplification products that occur when the extension times are significantly increased to capture longer ePAT products in this panel. The *GAL7* and *APQ12* amplicons (24 and 26 cycles of amplification, respectively) are included to show the specificity of endogenous galactose induction (*GAL7*) and an equal input of cDNA (*APQ12*).

The *CYC1* 3′ UTR and terminator is one of the most common terminating sequences used in yeast expression plasmids. In an experiment aimed to probe the function of the deadenylase Ngl3 we discovered that the *CYC1* 3′ UTR sequence generates abundant antisense transcripts. We made plasmids to overexpress *NGL3* from the *GAL1* promoter, with a cloning strategy where the *NGL3* gene could orient either in the forward or reverse orientation into the pGAL plasmid (Figure 1A). The latter was included in our experiments to control for secondary effects of overexpression, colloquially referred to as squelching. To confirm *NGL3* overexpression, we performed PCR based Poly(A)-Test (PAT) 3′ RACE assays on RNA isolated from cells before and after galactose induction. A version of the PAT assay, called ePAT and its sister assay TVN-PAT, are assays for detection of adenylated 3′ ends of RNA with either the full native poly(A)-tail or a minimal poly(A_12_)-tail, respectively (Figure 1B and (21)). Using a forward primer 59 bases upstream of the endogenous stop codon of *NGL3*, we noticed that in addition to the expected mRNA driven from the *GAL1* promoter, abundant *NGL3* ePAT amplicons were expressed from the reverse orientation plasmid (Figure 1C). This suggested that a second downstream promoter was driving expression of the *NGL3* transcript and that this utilized the *GAL1* promoter region for a PolyAdenylation Signal (PAS) and 3′ UTR. Sanger sequencing of this amplification product revealed that cleavage and polyadenylation occurred at −176 bases upstream of the Gal1 start codon (supplemental file 1).

We wondered if a cryptic promoter within the *CYC1* 3′ UTR might have driven transcription of the full-length *NGL3* transcript in this reverse orientation. Being fully in reverse complement, such a transcript would contain no premature stop codons or other obvious triggers of decay. Whereas any other antisense transcription from this 3′ UTR would be expected to hit numerous such sites and be rapidly destabilized by nonsense mediated decay in the empty plasmid or *NGL3*-forward plasmid. To determine if antisense transcription did indeed proceed from within the *CYC1* 3′ UTR and termination sequence, we designed an additional primer + 61 bases after the native *CYC1* stop codon but in reverse complement. A strong signal (second panel) from the empty plasmid, the *NGL3*-forward plasmid, and the *NGL3*-reverse plasmid confirmed that abundant antisense transcripts proceeded from the *CYC1* 3′ UTR. The site of 3′ cleavage and adenylation was different in each case (∼310, ∼330 and ∼2000 bases beyond the position of the primer), the adenylation sites presumably reflecting the different DNA sequences upstream of the *CYC1* 3′ UTR region in each plasmid. Galactose mediated induction of forward transcription did not appear to influence the level of reverse transcription products generated from this multicopy (2 micron) plasmid. This was illustrated in the pGAL-NGL3-F samples where forward transcription was strongly induced by galactose, but the level of the ∼300 bp antisense product driven from the cryptic promoter in the 3′ UTR remained at a constant level (Figure 1C).

Tiling arrays provide evidence for an endogenous antisense Stable Unannotated Transcript (*SUT641*) emanating from the *CYC1-UTR1* intragenic region (8). This suggests that transcriptional activity from this region is likely a property inherited from the native *CYC1* locus. Moreover, in our own recent genome-wide data for 3′ end-dynamics (20) there were clear antisense peaks of adenylation in the *CYC1*-*UTR1* intragenic region (Supplemental Figure S1A). Interestingly, the convergent transcription and overlap of the 3′ UTRs of *CYC1* and *UTR1* was described in the earliest literature describing the use of this 3′ UTR (26). Albeit, it is not clear now if what was being measured was the overlap from a full-length *UTR1* transcript and its associated 3′ UTR or the activity of the *SUT641* promoter that derives from the intergenic region. In our hands, convergent antisense transcription from the *CYC1* 3′ UTR resulted in transcripts of similar abundance to the *bone fide* forward oriented transcript as judged by the cycles of amplification required for detection. Thus, we caution that studies that utilize expression plasmids terminating in the *CYC1* sequence hold the potential for significant and unexpected contributions by anti-sense transcription.

### GFP-tagged strains reveal promoter-dependent control of convergent 3′ UTR-driven transcription

**Figure 2.**
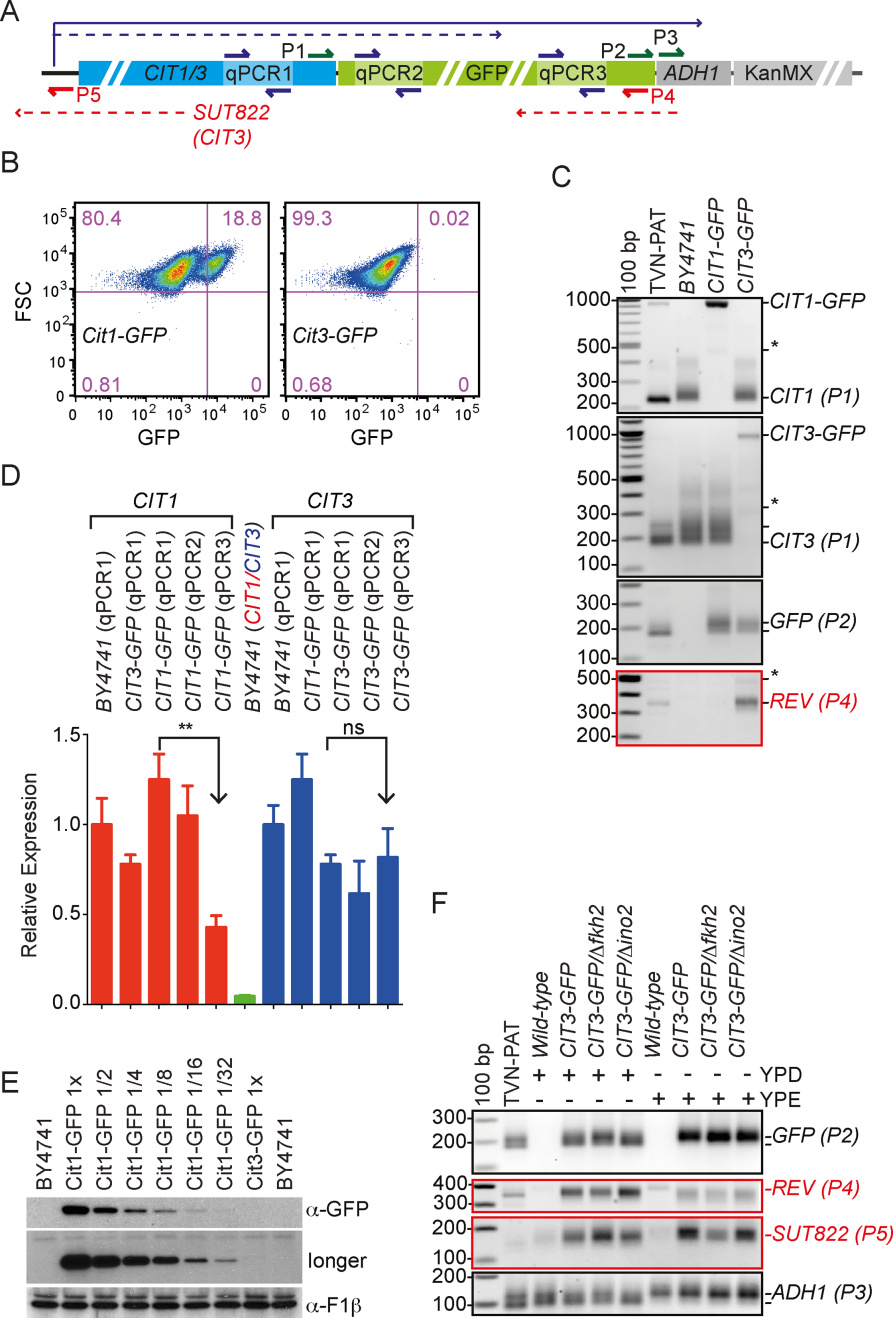
Locus specific antisense transcription from the *ADH1* 3′ UTR in GFP-tagged strains. (**A**) Schematic representation of the organization of the GFP-tagged library (3). The positions of ePAT and qPCR primers are indicated by green/red arrows or paired blue arrows, respectively. The solid or dashed lines indicate potential transcripts as in Figure 1. (**B**) Flow cytometry of stationary-phase Cit1-GFP and Cit3-GFP expressing strains. The ∼19% cells in the upper right-hand quadrant of the Cit1-GFP plot are the GFP expressing cells. In contrast there is no specific signal for Cit3-GFP expression (see also Supplementary Figure S2). (**C**) *BY4741*, *CIT1-GFP* or *CIT3-GFP* cells were grown to stationary phase in rich media and analysed by ePAT for forward and convergent antisense transcription. The TVN-PAT assay was prepared from pooled RNA from the three strains. The gene-specific P1 primers recognize either *CIT1* (top panel) or *CIT3* cDNA (second panel). The P2 primer anneals near the stop codon of GFP. The P4 primer amplifies any cDNA derived from antisense transcripts running out of the *ADH1* 3′ UTR sequence (red). Note the lower GFP mRNA level in the *CIT3-GFP* cells and the corresponding increase in convergent antisense transcription in this strain. (**D**) Quantitation of mRNA expression by qRT-PCR. In red, *CIT1* mRNA levels in the indicated strains was normalized first to actin and then to the *CIT1* level in the BY4741. The expression levels of the fusion-transcripts were detected by three different primers pairs (qPCR1, qPCR2 and qPCR3) as indicated in the schematic. The qPCR1 primer pair detects the gene of interest in tagged and untagged strains. The qPCR2 and qPCR3 primer pairs detect mRNA at the 5′ and 3′ ends of the GFP sequence, respectively (Note: this approach cannot discriminate sense from antisense contributions). In green, the ratio of *CIT1/CIT3* mRNA in the wild-type (BY4741) strain and normalized to the *CIT1* level in the wild-type strain is shown to illustrate the much reduced expression of *CIT3* compared to *CIT1*. In blue, *CIT3* mRNA was analysed as above in the indicated strains with the indicated primer pairs and was normalized to the average *CIT3* level in wild-type. Error bars reflect SEM n = 4 and *P*-values (** = 0.0019) were generated using unpaired parametric t-tests (prism software). (**E**) Protein lysates were prepared from either the wild type (BY4741) strain or from the two GFP-tagged strains. Dilutions of the Cit1-GFP strain were prepared by mixing with the indicated proportions of lysate from the untagged BY4741 strain. The Cit3-GFP and BY4741 lysates were loaded undiluted. GFP was detected using a commercial monoclonal antibody and an anti-mouse HRP conjugated secondary antibody. The two panels show 5 and 40-min exposures, respectively. The longer exposure gives ∼4-fold increase in detection sensitivity (i.e. band intensity of the 1/32 dilution at 40 min is approximately equivalent to the 1/8 dilution after 5 min exposure). The bottom panel is the same blot stripped and re-probed with an antibody recognizing the F1β subunit of the mitochondrial ATPase as a loading control. (**F**) The regulation of the *CIT3* locus in response to ethanol induction of transcription and loss of the transcriptional regulators Fkh2 and Ino2. *CIT3-GFP* cells were grown in rich fermentative (YPD) or respiratory (YPE) media containing either glucose or ethanol as the carbon source. Growth in respiratory media resulted induction of *CIT3* transcription. Detection of the GFP portion of the fusion mRNA by use of the P2 primer indicates the use of a more distal adenylation site in the *ADH1* 3′ UTR in association with transcriptional up-regulation. This adenylation site switching is also seen in the endogenous *ADH1* locus (P3; bottom panel). Transcriptional induction results in down-regulation of the *CIT3-GFP* convergent antisense transcript detected by P4 (in red). Loss of Δ*fkh2* toggles forward and reverse transcription. The 3′ –processing of SUT882 (antisense to *CIT3* see schematic) is shifted by transcriptional induction of *CIT3* and is sensitive to the presence of the GFP epitope (third panel).

A second commonly utilized terminating sequence in yeast cell biology is the *ADH1* 3′ UTR. Analysing our PAT-seq data (20) we noticed that this locus also contained evidence for convergent transcription proceeding from the tail-to-tail oriented gene *MHF1*, the 3′ UTR of which overlaps the *ADH1* 3′ UTR by ∼80 bases (Supplemental Figure S1B). Albeit in this case we found no prior evidence for a promoter activity in the 210 bases of *ADH1-MHF1* intragenic sequence. Nonetheless, we asked if this 3′ UTR inherited a propensity for overlapping 3′ UTRs when utilized in heterologous contexts, and turned to the GFP-tagged collection (3). The organization of the tagging cassette is illustrated in Figure 2A. We designed a series of primers around features of two such strains we had on hand (*CIT1*-GFP and *CIT3-*GFP) as indicated. Previous work from our lab and by others showed that expression of both *CIT1* and *CIT3* was induced by starvation (24,27), but this induction was clearly to very different levels. Whereas flow cytometry of cells grown to saturation in rich media shows a clear population of GFP-positive cells in the *CIT1*-*GFP* strain (24), Cit3-GFP fluorescence was not detected in any cells by this method (Figure 2B; see also Supplemental Figure S2). Primers designed near the endogenous stop-codon of *CIT1* or *CIT3*, indicate that the GFP-fusions result in low-level internal truncations (indicated by asterisk) in addition to the major full-length transcript (Figure 2C first two panels). Most interestingly, the lowly expressed full-length *CIT3*-GFP fusion (third panel), was associated with strong antisense transcription (bottom panel). Such antisense transcription is not normally monitored, and thus remains unaccounted for by standard measures of gene expression. To ask if it quantitatively influenced apparent forward transcription, we performed quantitative reverse transcription PCR (qPCR) with primers designed towards 3 positions across the GFP fusion as indicated by the schematic (Figure 2A). If the truncation products quantitatively reduce the amount of full-length product, this should be seen as a reduction in the normalized signal for each primer pair qPCR1 > qPCR2 > qPCR3. Unless of course, abundant antisense transcripts also added significantly to the signal, in which case qPCR3 would be expected to increase in *CIT3-GFP* compared to *CIT1-GFP*. The RNA (from Figure 2C) was reanalysed by qPCR. The data are presented relative to the average expression of either of *CIT1* or *CIT3* in untagged wild-type strains (Figure 2D red and blue bars, respectively). This normalization removes the ∼21-fold higher expression level of *CIT1* over *CIT3* (Figure 2D green bar), but highlights a significant reduction in signal 5′-to-3′ in the *CIT1* transcript, this is not observed for the *CIT3* transcripts, and is presumably explained by the contribution of convergent antisense RNA to the overall qPCR signal.

In contrast to the flow cytometry (Figure 2B), and fluorescence microscopy (data not shown) both ePAT and qPCR suggest that the *CIT3* mRNA was expressed, if at significantly lower levels than *CIT1*. Sequencing of the full *CIT3-GFP* fusion confirmed locus integrity and an absence of mutations or other genomic rearrangements that would limit protein expression (data not shown). Therefore, to determine if the protein was expressed, but at levels too low for fluorescence visualization, we performed western blot analysis comparing the accumulation of Cit1-GFP and Cit3-GFP. To capture the expression difference indicated by quantitation of mRNA abundance (Figure 2C) Cit1-GFP protein lysate was diluted with wild-type (no GFP) lysate to the indicated levels (Figure 2E). By longer exposure, the 1/32 dilution of Cit1-GFP was clearly visible but Cit3-GFP failed to be detected. Estimating the difference in band intensity between the two exposures, we predicted that our detection limit was equivalent to a ∼1/128 dilution. But, that if it was translated at all, the Cit3-GFP fusion was accumulated at levels lower than this.

To our knowledge, there is little precedent for antisense inhibition of translation in yeast. Yet the clear relationship between the low expression of Cit3-GFP and expression of an antisense RNA suggested that 5′-end mediated transcriptional repression at this locus in glucose media was somehow impacting regulation at the 3′ -end. In other words, we speculated that 5′ -end mediated repression was influencing a cryptic promoter in the *ADH1* 3′ UTR to induce silencing antisense transcription. We therefore searched for conditions that induce *CIT3* expression. The tiling arrays of Xu *et al*. (2009) suggested a carbon-source dependent inverse relationship between *CIT3* expression and convergent transcription of *SUT822*, a non-coding RNA that initiates from a cryptic promoter ∼2/3rd into the *CIT3* open reading frame (see schematic in Figure 2A and (8)). The literature additionally reports negative regulation by Fkh2 (28) and holds evidence for Ino2 binding in the *CIT3* locus by chromatin immunoprecipitation (29). We introduced both these mutations into the *CIT3*-GFP strain.

To ask if rewiring of the *ADH1* 3′ UTR reflected an extension to native induction of antisense transcription by transcriptional repression, we grew the *CIT3-GFP* strains as indicated in media with either glucose (YPD) or ethanol (YPE) as a carbon source (Figure 2F). This confirmed up regulation of *CIT1-GFP* in ethanol media and suggested a modest derepression of *CIT3-GFP* in the Δ*fkh2* mutant. The induction of *CIT3-GFP* was associated with selection of a distal, polyadenylation site. This choice favouring a longer 3′ UTR is a feature of the native *ADH1* locus (bottom panel) and is also observed in the untagged *CIT3* gene (data not shown). As predicted, the induction of *CIT3-GFP* expression reduced the expression of the cryptic antisense transcript (second panel) and the increase in forward transcription in the Δ*fkh2* mutant was associated with a decrease in reverse transcription. Finally, placement of a primer (P5) to detect the last ∼150 bases of the endogenous *SUT822* non-coding RNA (see Figure 2A), showed that although the overall abundance was not changed by induction of its sense counterpart *CIT3*, the 3′ -end processing was changed to favour a more distal polyadenylation site. It is important to note that the introduction of the GFP-Fusion cassette, a significant distance downstream of the cryptic SUT882 promoter, resulted in a major up regulation (compare wild-type and CIT3-GFP strains). The adenylation sites for *SUT822* captured here were ∼75 bases upstream of the *CIT3* start codon. CUTs and SUTs often display heterogeneous 5′ and 3′ ends (7), and it is likely that additional changes occur for this SUT that are not captured by the placement of our P5 primer. The change in level of the *SUT822* in response to the Δ*fkh2* mutation, further suggest that its expression is intimately linked to the transcriptional landscape of the full *CIT3* locus.

### TAP-tagging induces 3′ truncated transcripts by locus dependent alternative polyadenylation

**Figure 3.**
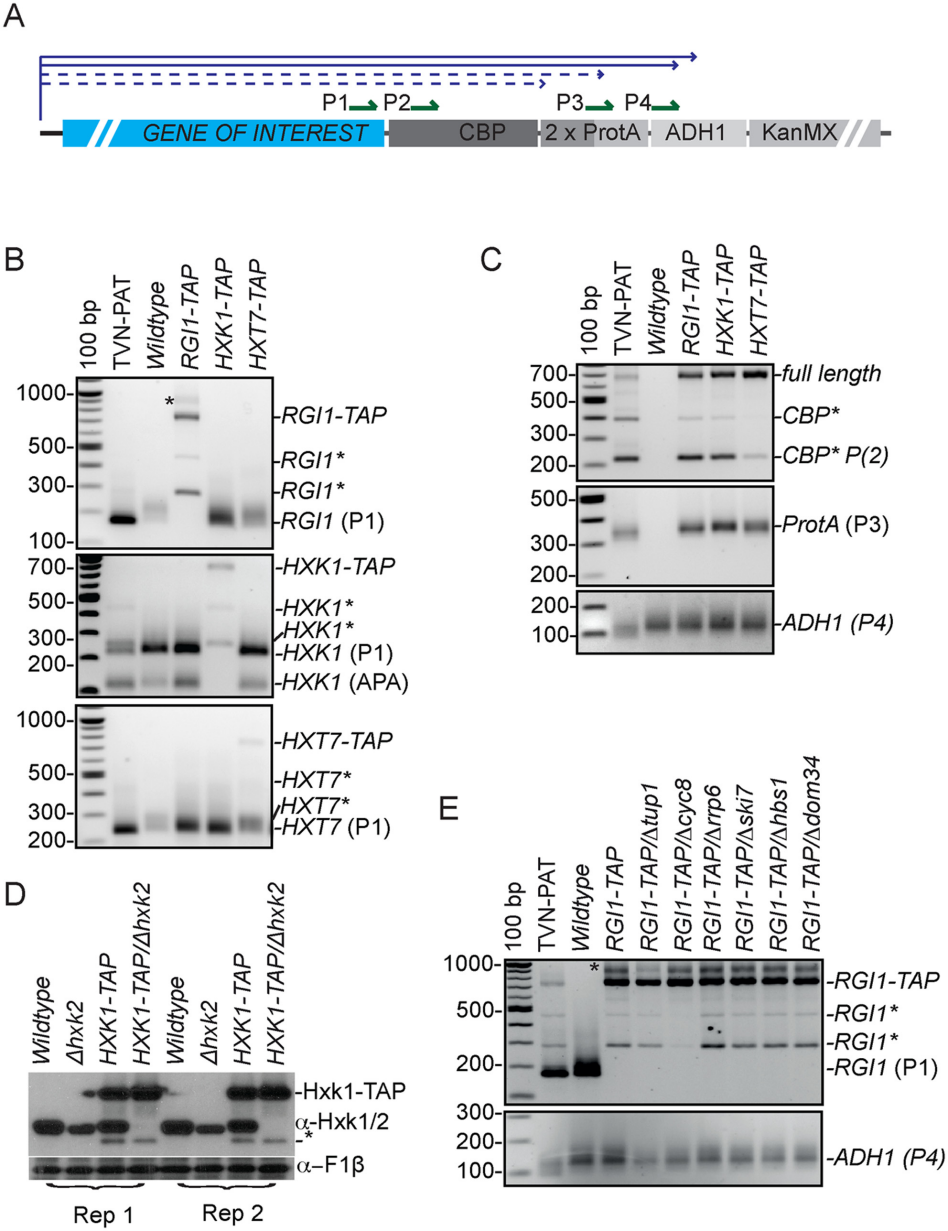
Locus-dependent aberrant 3′-end formation and antisense transcription in TAP-tagged strains. (**A**) Schematic representation of the integration cassette used to generate the TAP-tagged library (2). The solid blue line and arrowhead indicate the full-length forward transcript and position of correct cleavage and adenylation. The dashed blue lines indicate the potential premature 3′-end-formation sites. (**B**) The *BY4741*, *RGI1-TAP*, *HXK1-TAP* and *HXT7-TAP* strains were grown in respiratory media to an OD_600_ of ∼0.6 to induce target gene transcription, and analysed by ePAT assays. TVN-PAT assays were prepared from a pool of the four separate RNA samples. Gene-specific (P1) primers were used to amplify across the cassette to the poly(A)-tail. Multiple amplicons of discreet lengths indicate alternative adenylation sites. *HXK1*, presents with mRNA having two APA isoforms. The asterisk (*) beside the names indicates likely truncation products. The gene name-TAP indicates the full-length fusion. An asterisk (*) next to bands on the gel image indicate non-specific amplification induced by longer extension times. (**C**) To determine if the TAP tag contained universal alternative adenylation sites, the same cDNA analysed in Figure 2B was amplified using the universal P2 primer that recognizes the start of the CBP segment in the top panel. In the lower panel the P3 primer binds at the junction of the two protein-A sequences. (**D**) Cellular Hexokinase levels were detected by immunoblotting with an anti-Hxk1 polyclonal antibody that recognizes both the Hxk1 and Hxk2 proteins. Wild-type yeast, Δ*hxk2*, *HXK1-TAP* and *HXK1-TAP*/Δhxk2 strains were prepared in biological replicate. The slower migrating band corresponds to the fusion-protein. The same blot was stripped and re-probed with an antibody recognizing the F1β subunit of the mitochondrial ATPase. The -* indicates a truncation product or decay intermediate. Note that this product is too small to be a direct protein product of mRNA truncations identified in panel 3C. (**E**) The prematurely truncated mRNA appear to derive from alternative nuclear 3′-end processing rather than translation-dependent decay mechanisms. The wild-type *BY4741* strain and the indicated mutations of the *RGI1-TAP* strains were grown in respiratory media (YPEG) to an OD_600_ of ∼0.6 to induce target gene transcription, and analysed by ePAT assays. TVN-PAT assays were prepared from a pool of all indicated RNA samples. The first three mutants (Δ*tup1*, Δ*cyc8*, Δ*rrp6*) are implicated in nuclear transcription and RNA metabolism whereas the last three (Δ*ski7*, Δ*hbs1*, Δ*dom34*) have functions in translation dependent mRNA decay.

We had on hand an additional series of TAP-tagged strains (*RGI1-TAP*, *HXK1-TAP* and *HXT7-TAP*) from previous work (30). To ask if introduction or the TAP-epitope tag was also associated with unexpected transcriptional activity, the strains were grown in respiratory media (YPEG) to induce target gene-expression. We designed PAT primers to each of the genes of interest (P1; Figure 3A). Based on the position of the primer, the sequence of the tag and the position of adenylation within the *ADH1* 3′ UTR, we expected PCR amplicons of 704, 708 and 750 bases for *RGI1-TAP, HXK1-TAP* and *HXT7-TAP*, respectively. Contrary to this expectation, multiple premature adenylation sites appeared to be utilized in each strain tested (Figure 3B).

To confirm the identity of the truncation products and to determine the exact position of the adenylation sites, two additional primers were designed towards elements universal to the TAP-tagged strains; the start of the Calmodulin Binding Protein (CBP) sequence, and straddling the duplicated protein-A sequence (P2 and P3, respectively). Sanger sequencing of the 3 distinct amplicons that result from the P2 primer (Figure 3C), confirmed additional cleavage and adenylation sites at the start of each of the duplicated protein-A sequences 210 bases and 384 bases from the start of the tag. Two closely spaced distal polyadenylation sites were in the correct position compared to the adenylation site of *ADH1* in its native configuration (669 bases into tag). The amplicon intensity representing each of the different 3′ UTR-isoforms appeared to be strain specific, suggesting that features within the local genomic landscape influence the steady-state level of these truncated products. That is*, RGI1-TAP* was biased towards accumulation of the shortest truncating-isoform whereas *HXT7-TAP* is biased towards the full-length transcript correctly processed from cleavage and adenylation within the *ADH1* 3′ UTR.

A surprisingly high proportion of the forward transcription resulted in premature cleavage and polyadenylation prior to the TAP tag in the strains tested. The tight gel migration of ePAT products for the first two APA products suggested that these RNA were terminated by only short poly(A)-tails. The sequencing supported this, with the average sequenced tail of the truncated mRNA being 12 A-residues (n = 8), whereas the average sequenced length for the correct distal-most adenylation site was 38 A-residues (n = 2). Short poly(A)-tails such as these are associated with non-coding RNA, transcriptional-stall products and decay intermediates (31). Because translation of such truncation products (without the tag) could distort interpretation of experiments where quantitation is important, we asked if a truncated Hxk1-TAP transcript was translated. Hxk1 has a homolog (Hxk2) that cross-reacted with our antibody, so we first generated a Δ*hxk2/HXK1-TAP* strain to search for expression of the hypothetical tagless-Hxk1 (Figure 3D). We could not detect such a protein, thus if the truncated mRNA did engage cytoplasmic ribosomes, associated polypeptides were not stably accumulated. Indeed transcripts without stop codons are likely to invoke non-stop decay (32), and any proteins produced would terminate in poly-lysine (encoded by the A-tail) and would thus likely be substrates for proteasome-mediated decay (33).

There are at least two plausible mechanistic sources for the short poly(A)-tailed 3′ truncated transcripts. They could be products of transcription generated by induction of premature cleavage and adenylation. Alternatively they could be generated during failed translation by activation of cytoplasmic surveillance such as no-go or non-stop decay (34,35). To begin to stage the step(s) in RNA metabolism that give rise to the truncations, we introduced a series of mutations into the *RGI1*-TAP strain. The literature provides evidence for transcriptional regulation of the *RGI1* locus by the general transcriptional repressor Tup1/Cyc8 (28,29). To additionally probe involvement of the cytoplasmic exosome, and decay by non-stop and/or no-go translation we introduced the Δ*ski7*, Δ*hbs1* and Δ*dom34* mutations, respectively. Finally, the *Δrrp6* mutation was introduced to determine if the truncation products were subject to nuclear surveillance (Figure 3E). Whereas our data provide evidence for an involvement of nuclear RNA metabolism in the generation of the truncated forms of the *RGI1*-TAP transcript, the cytoplasmic functions we tested do not appear to influence accumulation of the truncated transcripts. Curiously, loss of either Tup1 or Cyc8 seems to suppress alternative truncating polyadenylation. In contrast, loss of Rrp6, the nuclease component of the nuclear exosome, results in a slight increase in the level of the truncated transcript consistent with a failure of quality control for this transcript and thus nuclear turnover. If significant proportions of the truncated transcripts had been exported from the nucleus and had engaged cytoplasmic ribosomes, the signal would also be expected to have increased in the *Δski7*, *Δhbs1* and *Δdom34* mutants. As this was not the case, we conclude that the truncations were generated during transcription by premature cleavage and adenylation.

## DISCUSSION

Our data promote the idea that transcriptional control imposes processing decisions for the entire genomic locus. Starting, as expected, at the level of transcription, but extending to adenylation-site choice within the 3′ UTR, and to the induction of convergent antisense transcription. We show that antisense-transcripts are induced from cryptic promoters in response to controlling upstream promoter decisions. The evidence for this in our data is provided by the universal *ADH1* 3′ UTR that terminates the TAP and GFP tagged strains used in this study. The adenylation-site choice and antisense induction from the *ADH1* 3′ UTR is locus specific (Figures 2C and 3C), and therefore regulation must be imposed by the local genomic landscape. We propose that such 5′ mediated control over 3′ UTR dynamics is reinforcing to transcriptional control. This notion extends current models of regulatory control by convergent transcription. For example, in the classic case of the meiosis-specific *IME4* gene, silencing is achieved by transcription of a ncRNA *IME2*, initiated from within the *IME4* 3′ UTR. Binding of the a1-α2 repressor complex upstream of *IME2* in diploid cells is thought to be independently controlled, and limits *IME2* expression to allow forward transcription of *IME4* in a classic self-regulatory loop (36,37). Yet forced overexpression of *IME4* in haploid cells by introduction of the *GAL1* promoter overrides any convergent antisense transcription at this locus (38). This may be a simple case of transcriptional dominance by a strong promoter suppressing weaker convergent transcription by polymerase collision, but may also derive from the promoter driven rewiring of 3′ dynamics.

Promoter driven rewiring of 3′ -end formation and activation of cryptic promoters does not always appear to be as simple as a regulatory negative feedback loop however. Unlike the situation at native genomic loci; driven from a multi-copy plasmid, induction of abundant forward transcription from the inducible *GAL1* promoter does not directly influence the steady-state level of convergent antisense transcripts (Figure 1A). This argues against transcriptional collision suppressing reverse transcription from the *CYC1* cryptic promoter. This might be explained in terms of populations, with transcriptional direction being fixed on individual plasmid templates or within individual cells within populations.

It is not yet clear what mechanistically differentiates the early metabolism of coding from of non-coding RNA. The outcome of cryptic transcription from the *CYC1* 3′ UTR is very different when the full-length *NGL3* ORF is transcribed (pGal-NGL3-rev) in comparison to the non-coding transcripts that run antisense to either the empty plasmid or the forward oriented *NGL3* gene (Figure 1C). Whereas the *CYC1* driven *NGL3* transcript is abundant, long poly(A)-tailed and presumably stably translated in the cytoplasm, the nonsense antisense products are slightly less abundant, short-tailed, and likely subject to rapid decay after synthesis. The transcription initiation rates from the cryptic promoter are not expected to be different in the different plasmids, but there must exist an early metabolic branch-point with built-in recognition of RNA that encodes protein from the RNA that does not.

The close physical association of promoters with sites for 3′ -end processing by gene loops provides a convenient mechanism for RNA polymerase II recycling, and in general for the insulation of the regulation of individual genomic loci (39). There is an emerging picture that suggests that properties surrounding transcription can influence the 3′ UTR choice (40–42). For example, Ji *et al.*, (2011) show both in human genome-wide data and gene-by-gene plasmid-based experiments that transcriptional activity influences 3′ UTR choice (40). Albeit, unlike our data shown here (Figure 2F) where transcriptional induction results in the switch to a longer 3′ UTR, they report a general trend towards longer 3′ UTRs correlating with lower expression. Others have also noted exceptions to this generalization. In *Drosophila*, e.g. the rate of transcription elongation controls adenylation-site choice in the transcription factor Polo, with slower elongation favouring a proximal site for adenylation (43). It will require further study to determine if these conflicts are due to species differences, or real mechanistic differences in the control of processing. Our data suggest that the genome-wide collections of tagged yeast strains could provide an excellent resource for probing these differences.

The induction of truncating 3′ end formation at the start of each of the duplicated Protein A moieties of the TAP tag (Figure 3), while not being of serious concern for most protein studies, is problematic for studies of gene expression. If, as our data show, the extent of inappropriate truncation is locus dependent and thus unpredictable, it may well introduce unacceptable variability across strains. The truncations occur after an AU rich region (71% AU in the 35 bases proceeding the polyadenylation site) as do native polyadenylation sites. That adenylation sites are so easily inadvertently introduced by researchers with well intended cloning strategies, is of concern for studies where mRNA expression is correlated to protein expression, or where the native 3′ UTR is manipulated, e.g. by introduction of MS2 recognition sequence to localize mRNAs (44). Indeed during revision of this manuscript, it was reported that MS2 sequences stabilize mRNA and result in decay resistant 3′ fragments that can distort interpretation of localization data (45). Thus, highlighting an additional complication in the interpretation of data based on genetic modification of native mRNA expression.

One caveat to the interpretation of inappropriate truncating adenylation is that *bone-fide* 3′ -end formation is not easily distinguished from 3′ -ends generated by the adenylation that is associated with mRNA surveillance, or stalling of translation and associated stimulation of no-go decay (35). We also noticed a propensity for transcript truncation when a single Protein-A tag was introduced into genomic loci using an alternative strategy of epitope tagging ((46) data not shown). Therefore the property of inappropriate adenylation is likely intrinsic to the Protein A sequence rather than associated with the upstream Calmodulin binding protein moiety or the fact that the tag is duplicated. Our data suggested that the source of truncation in the strains reported here was due to nuclear activity. Thus, as with the finding that induction of antisense transcription is driven by the upstream promoter, this feature of locus dependent truncating cleavage and adenylation provides an excellent model for the study of alternative adenylation machineries, including that imposed by the Nrd1 and the TRAMP complex.

In sum, our data provide yet another novel application for the yeast genomic resource of TAP and GFP tagged libraries. That is, as new models to understand the regulatory paradigms for promoter driven control of 3′ dynamics, antisense convergent transcription and 3′ -end formation.

## Supplementary Material

SUPPLEMENTARY DATA
