# Supplementary material for: Epitope-tagged yeast strains reveal promoter driven changes to 3′-end formation and convergent antisense-transcription from common 3′ UTRs
Source: Nucleic Acids Res. 2015 Oct 19;44(1):377–86. doi: 10.1093/nar/gkv1022 (PMC4705644; doi:10.1093/nar/gkv1022)
Supplement: SUPPLEMENTARY DATA [file supp_44_1_377__index.html]

Epitope-tagged yeast strains reveal promoter driven changes to 3′-end formation and convergent antisense-transcription from common 3′ UTRs — SUPPLEMENTARY DATA 

# Epitope-tagged yeast strains reveal promoter driven changes to 3′-end formation and convergent antisense-transcription from common 3′ UTRs

## SUPPLEMENTARY DATA

- SUPPLEMENTARY DATA
- SUPPLEMENTARY DATA
