# Supplementary material for: Epitope-tagged yeast strains reveal promoter driven changes to 3′-end formation and convergent antisense-transcription from common 3′ UTRs
Source: Nucleic Acids Res. 2015 Oct 19;44(1):377–86. doi: 10.1093/nar/gkv1022 (PMC4705644; doi:10.1093/nar/gkv1022)
Supplement: SUPPLEMENTARY DATA [file supp_gkv1022_nar-01607-v-2015-File005.pdf]

Supplemental Data Files

Supplemental Table 1. Contains the details of all cloning, ePAT and qPCR oligonucleotide primers. Where applicable, sequencing data for adenylation sites is also provided.

Supplemental Figure 1. Native antisense and convergent transcription generate 3' overlap. These data are taken from Harrison et al., a manuscript currently *in press* at RNA MS ID#: RNA/2014/048355

Harrison, P.H., Powell, D.R., Clancy, J.L., Preiss, T., Boag, P.R., Traven, A., Seemann, T. and Beilharz, T.H. (2015) PAT-seq: *A method to study the integration of 3' UTR dynamics with gene-expression in the eukaryotic transcriptome*. *RNA*, (*in press*).

(**A**) Integrated Genome Browser snapshot of reads aligned to the intergenic region *CYC1-UTR1* the green and blue peaks represent reads aligning to the forward and reverse strand respectively. The RNA and its adenylation is represented in schematic. The data are taken from Harrison et al (2015) and are available for interactive viewing here [http://rnasystems.erc.monash.edu/]. The tracks names indicate the following: Ambiguity, if present (red) would mean that there were a danger of ambiguous mapping to the genome based on non-unique sequence. Poly(A)-For and/or Poly(A)-Rev means that adenylation was detected a the indicated sites (in purple) the height of the peak correlates to the number of reads that terminated at the given poly(A)-site. Multiple peaks mean sites of alternative adenylation or micro heterogeneity (red dashed lines in schematic). All logrep1 and all logrep2 refer to two biological repeats of BY4741 cells grown in rich media to mid-log phase in green are all aligned reads that mapped to the forward direction, and in blue all reads aligned to the reverse strand.

(**B**) Integrated Genome Browser snapshot as above showing reads aligned to the intergenic region *ADH1-MHF1.*

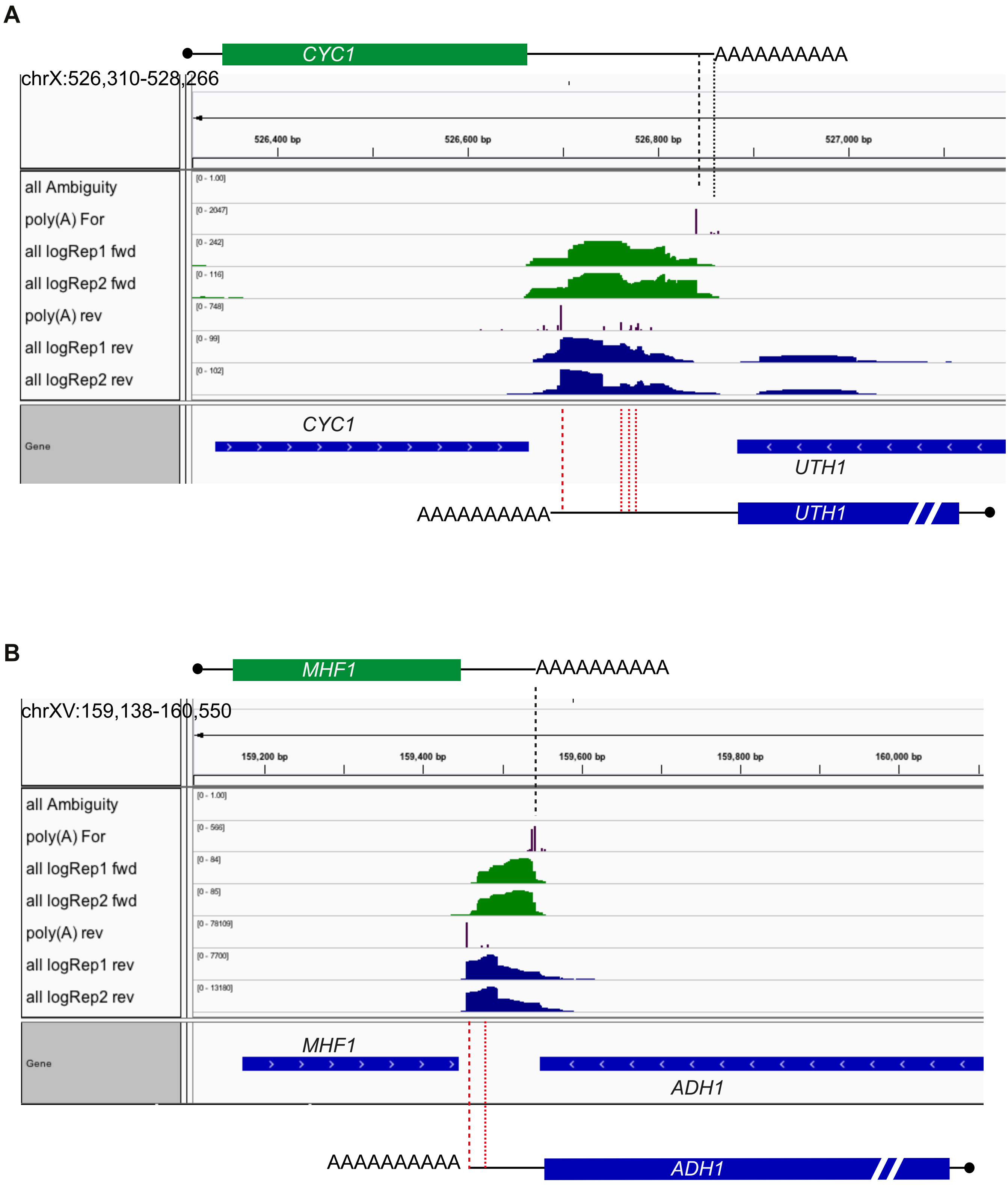

A
CYC1
AAAAAAAAAA
chrX:526,310-528,266
526,400 bp
526,600 bp
526,800 bp
527,000 bp
all Ambiguity
poly(A) For
all logRep1 fwd
all logRep2 fwd
poly(A) rev
all logRep1 rev
all logRep2 rev
[0 - 1.00]
[0 - 2047]
[0 - 242]
[0 - 116]
[0 - 748]
[0 - 99]
[0 - 102]
Gene
CYC1
UTH1
AAAAAAAAAA
UTH1
B
MHF1
AAAAAAAAAA
chrXV:159,138-160,550
159,200 bp
159,400 bp
159,600 bp
159,800 bp
160,000 bp
all Ambiguity
poly(A) For
all logRep1 fwd
all logRep2 fwd
poly(A) rev
all logRep1 rev
all logRep2 rev
[0 - 1.00]
[0 - 566]
[0 - 84]
[0 - 85]
[0 - 78109]
[0 - 7700]
[0 - 13180]
Gene
MHF1
ADH1
AAAAAAAAAA
ADH1

Supplemental Figure 2. Flow cytometry of yeast cells to show expression of GFP fusions.

The 6 panels represent the indicated yeast stains grown to saturation in YPAD in biological replicate. The x-axis indicates the level of GFP fluoresce intensity and on the y-axis FSC-A represents the approximate size of the cells

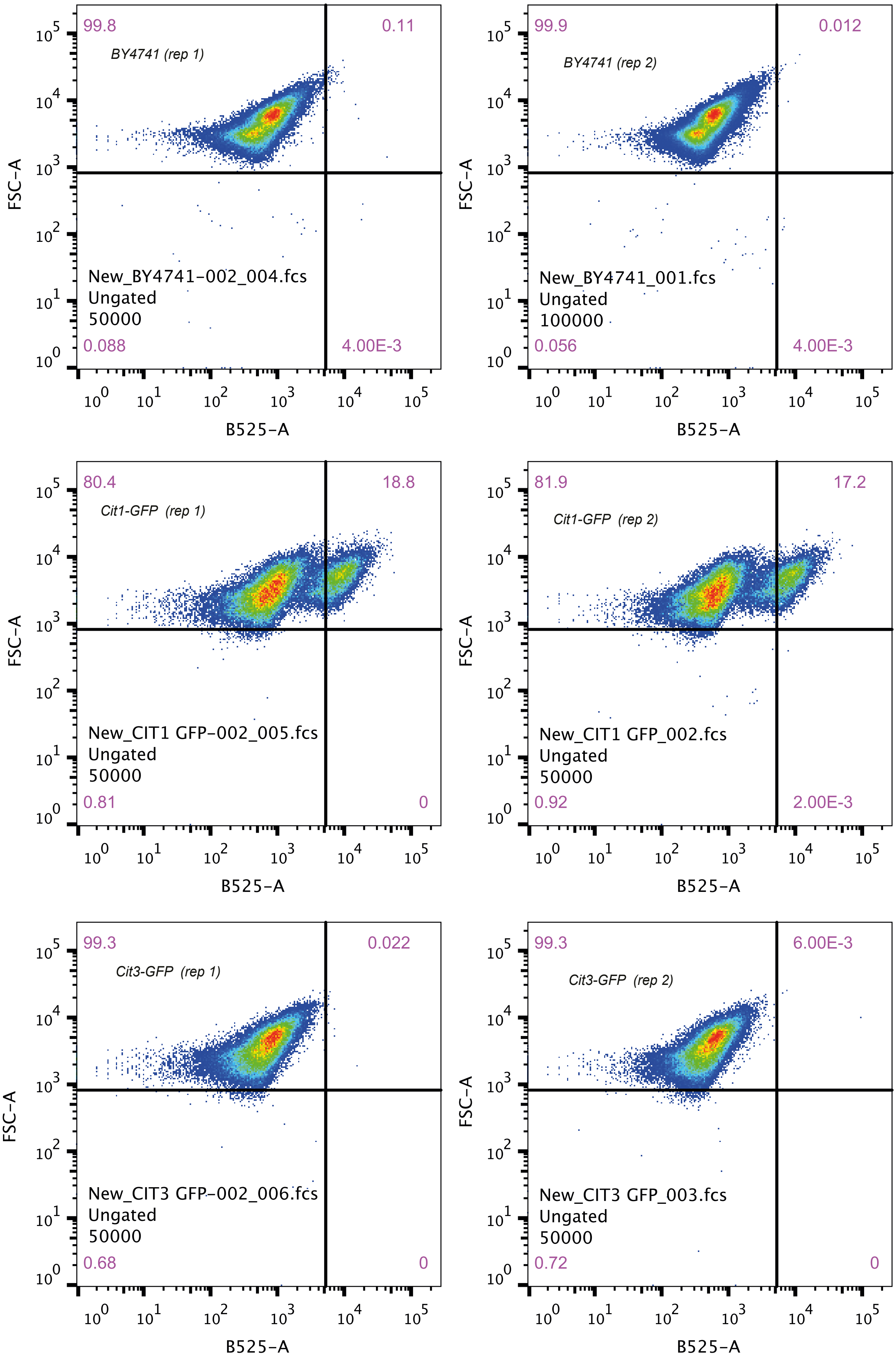
99.8
0.11
BY4741 (rep 1)
New_BY4741-002_004.fcs
Ungated
50000
0.088
4.00E-3
FSC-A
B525-A
99.9
0.012
BY4741 (rep 2)
New_BY4741_001.fcs
Ungated
100000
0.056
4.00E-3
80.4
18.8
Cit1-GFP (rep 1)
New_CIT1 GFP-002_005.fcs
Ungated
50000
0.81
0
81.9
17.2
Cit1-GFP (rep 2)
New_CIT1 GFP_002.fcs
Ungated
50000
0.92
2.00E-3
99.3
0.022
Cit3-GFP (rep 1)
New_CIT3 GFP-002_006.fcs
Ungated
50000
0.68
0
99.3
6.00E-3
Cit3-GFP (rep 2)
New_CIT3 GFP_003.fcs
Ungated
50000
0.72
0
